# Supplementary material for: Broad clinical manifestations of polygenic risk for coronary artery disease in the Women’s Health Initiative
Source: Commun Med (Lond). 2022 Aug 25;2:108. doi: 10.1038/s43856-022-00171-y (PMC9411562; doi:10.1038/s43856-022-00171-y)
Supplement: Supplementary file 1 — Description of Additional Supplementary Files [file 43856_2022_171_MOESM1_ESM.pdf]

Description of Additional Supplementary Files

File Name: Supplementary Data

Description: Complete summary statistics of the WHI association analyses
