# Supplementary material for: Broad clinical manifestations of polygenic risk for coronary artery disease in the Women’s Health Initiative
Source: Commun Med (Lond). 2022 Aug 25;2:108. doi: 10.1038/s43856-022-00171-y (PMC9411562; doi:10.1038/s43856-022-00171-y)
Supplement: Supplementary file 2 — Supplemental Material [file 43856_2022_171_MOESM2_ESM.pdf]

Supplementary material

Broad Clinical Manifestations of Polygenic Risk for Coronary Artery Disease in the Women's Health Initiative

Shoa L. Clarke, MD, PhD,[1,2*] Matthew Parham, MS,[3*] Joanna Lankester, PhD,[1,2] Aladdin H. Shadyab, PhD,[4] Simin Liu, MD, ScD,[5] Charles Kooperberg, PhD,[6] JoAnn E. Manson, MD, DrPH,[7] Catherine Tcheandjieu, DVM, PhD,[8,9] Themistocles L. Assimes, MD, PhD[1,2,3]

[1]VA Palo Alto Health Care System, Palo Alto, CA, USA
[2]Department of Medicine, Division of Cardiovascular Medicine, Stanford University School of Medicine, Stanford, CA, USA
[3]Department of Epidemiology and Population Health, Stanford University School of Medicine, Stanford, CA, USA
[4]Herbert Wertheim School of Public Health and Human Longevity Science, University of California, San Diego, La Jolla, CA, USA
[5]Department of Epidemiology, Brown University, Providence, RI, USA
[6]Fred Hutchinson Cancer Research Center, Seattle, WA, USA
[7]Department of Medicine, Brigham and Women's Hospital, Harvard Medical School, Boston, MA, USA
[8]Gladstone Institute, San Francisco, CA, USA
[9]Department of Epidemiology and Biostatistics, University of California San Francisco, San Francisco, CA, USA
* These authors contributed equally

Corresponding Author
Themistocles L. Assimes, MD, PhD
1070 Arastradero Road, Suite 300, Palo Alto, CA 94304
Phone: 650-498-4154
tassimes@stanford.edu

Supplementary Table 1. Overview of Genetic Studies Used to Define Study Population

(WHI Study #)	Hip Fracture GWAS	GARNET	WHIMS+	GECCO	MOPMAP	Breast Cancer Post GWAS
	(BA3)	(M13)	(W63)	(AS224)	(AS264)	(M18)
**Study that funded GWAS**	WHI-BAA3 National Heart, Lung, and Blood Institute (NHLBI)	WHI-GARNET - National Human Genome Research Institute (NHGRI)	NHLBI	WHI-AS224 - National Cancer Institute (NCI)	WHI-AS264 - National Institute of Environmental Health Sciences (NIEHS) and Univ of North Carolina	M18 - NIH
**GWAS platform**	Illumina 550K and 610K	Illumina HumanOmni1-Quad v1-0 B	HumanOmniExpress Exome-8v1_B	Illumina 610 and Cytochip 370K	Affymetrix Gene Titan, Axiom Genome-Wide Human CEU I Array Plate	*llumina OncoChip*
**Design**	Case-control	Case-control (4 case groups)	Cohort	Case-control	Case-Control	Case-Control
**Phenotype for cases**	Hip Fracture	Type 2 Diabetes, Myocardial Infarction, Stroke, Venous Thrombosis	NA	Colorectal cancer	Ventricular Ectopy (ever)	Breast Cancer
**Other sample details**	NA	Hormone Therapy Clinical Trial	Hormone Therapy Clinical Trial	NA	Controls selected within centers, years, seasons and visit years in which cases originated	NA
**Self-identified race/ethnicity**	Mostly non-Hispanic white	Mostly non-Hispanic white	Non-Hispanic white	Non-Hispanic white	Non-Hispanic white	Non-Hispanic white
**Sample size**	3,690	4,883	5,687	2,493	3,069	9,553

Supplementary Table 2. Questions/forms used to identify subjects with a known or likely history of atherosclerotic cardiovascular disease (ASCVD) at baseline.

Source	Disease	Question
Form 2 - Eligibility Screening	Stroke ever	Did a doctor ever say that you had a stroke?
Form 2 - Eligibility Screening	Stroke last 6 months	Did you have a stroke in the last 6 months?
Form 2 - Eligibility Screening	TIA ever	Did a doctor ever say that you had a small stroke that lasted less than 24 hours? This is sometimes called a transient ischemic attack or TIA.
Form 2 - Eligibility Screening	TIA last 6 months	Did you have a TIA in the last 6 months?
Form 2 - Eligibility Screening	MI ever	Did a doctor ever say that you had a heart attack? This is sometimes called a coronary, myocardial infarction, or MI.
Form 2 - Eligibility Screening	MI last 6 months	Did you have a heart attack in the last 6 months?
Form 30 - Medical History	Cardiac arrest ever	Please mark the conditions or procedures below that a doctor said you had. Cardiac arrest (where your heart stopped and needed to be restarted)
Form 30 - Medical History	Coronary bypass surgery ever	Please mark the conditions or procedures below that a doctor said you had. Heart bypass operation or coronary bypass surgery for blocked or clogged arteries in you heart
Form 30 - Medical History	Angioplasty of coronary arteries ever	Please mark the conditions or procedures below that a doctor said you had. Angioplasty of the coronary arteries (opening the arteries of the heart with a balloon or other device, sometimes called a PTCA)
Form 30 - Medical History	Carotid endarterectomy/ angioplasty ever	Please mark the conditions or procedures below that a doctor said you had. Carotid endarterectomy or carotid angioplasty (operation for blockage or narrowing of the arteries in your neck)
Form 30 - Medical History	Aortic aneurysm ever	Please mark the conditions or procedures below that a doctor said you had. Aortic aneurysm
Form 30 - Medical History	Angina ever	Did a doctor ever say that you had angina (chest pains from a heart problem)?
Form 30 - Medical History	Pills for angina now	Do you now take pills for angina?
Form 30 - Medical History	Peripheral arterial disease ever	Did a doctor ever say that you had claudication or peripheral arterial disease (poor blood flow to the legs or blocked or narrowed arteries to the legs)? Do not include varicose veins or phlebitis.
Form 30 - Medical History	Angiography for PAD ever	For the above condition, have you ever had: Angiography (dye in the arteries of the legs)?
Form 30 - Medical History	Angioplasty for PAD ever	For the above condition, have you ever had: Angioplasty (balloon catheter to open blockage)?
Form 30 - Medical History	Surgery to improve flow to legs for PAD	For the above condition, have you ever had: Surgery to improve blood flow in your legs (do not include surgery for varicose veins)?
Form 30 - Medical History	CABG/PTCA Ever	Computed from Form 30, questions 3.1.4 and 3.1.5. Indicator for whether the participant has a history of either CABG or PTCA.

TIA = transient ischemic attack; MI = myocardial infarction; PAD = peripheral arterial disease; CABG = coronary artery bypass; PTCA = Percutaneous Transluminal Coronary Angioplasty

Supplementary Table 3. Cohort characteristics. The study cohort was selected from the group of genotyped participants with inferred similar genetic ancestry. Non-genotyped participants are shown for comparison.

Characteristic	Study Cohort, no ASCVD at enrollment (n = 21,863)	Genotyped participants with inferred similar genetic ancestry (N=24,693)	Non-genotyped participants, self-identified non-Hispanic white (N=109,037)
Age	65.3 ± 6.3	65.66 ± 6.9	63.10 ± 7.2
Self-identified non-Hispanic White	21,698 (99.2)	24,504 (99.2)	109,037 (100)
Clinical Trial Status	15,068 (68.9)	16,959 (68.7)	38,744 (35.5)
**Health traits**			
Total cholesterol	223.4 ± 42.9	223.3 ± 43.0	221.3 ± 42.9
LDL-C	139.7 ± 39.0	139.7 ± 39.0	135.6 ± 39.6
HDL-C	55.0 ± 14.3	54.6 ± 14.3	56.3 ± 15.4
Triglycerides	143.0 ± 83.3	145.4 ± 84.2	136.4 ± 82.1
Systolic Blood Pressure	126.0 ± 6.0	129.2 ± 18.1	126.6 ± 17.8
Body Mass Index	28.5 ± 6.0	28.2 ± 5.8	27.5 ± 5.7
**Parent study outcomes**			
Clinical MI	1,309 (6.0)	1,673(6.8)	3,832 (3.5)
Breast Cancer	4,928 (22.5)	5,425 (22.0)	6,241 (5.7)
Colon Cancer	910 (4.20)	1,010 (4.1)	1,380 (1.3)
Diabetes	2,168 (9.9)	2,610 (10.6)	10,035 (9.3)
Hip Fracture	2,167 (9.9)	2,575 (10.4)	1,628 (1.5)

ASCVD = atherosclerotic cardiovascular disease; LDL-C = low-density lipoprotein cholesterol; HDL-C = high-density lipoprotein cholesterol; MI = myocardial infarction

Supplementary Table 4. Associations between polygenic risk for coronary artery disease and incident adjudicated cardiovascular diseases without risk factor adjustment.

Outcome	Cases	Controls	OR	LCI	UCI	P value
Coronary revascularization	1,530	20,333	1.55	1.46	1.64	6.2E-53
PTCA	1,150	20,713	1.52	1.43	1.62	6.8E-39
Myocardial infarction	1,309	20,554	1.43	1.35	1.52	1.3E-31
Coronary heart disease	1,792	20,071	1.33	1.26	1.40	1.1E-26
CABG	501	21,362	1.56	1.42	1.71	1.8E-20
All angina	620	21,243	1.41	1.30	1.54	9.8E-16
Ischemic stroke	1,108	20,755	1.16	1.08	1.23	8.4E-06
All stroke	1,457	20,406	1.14	1.07	1.20	1.1E-05
Carotid disease	293	21,570	1.20	1.06	1.35	3.0E-03
Peripheral artery disease	275	21,588	1.21	1.06	1.37	3.2E-03
Congestive heart failure	579	21,284	1.09	1.00	1.19	6.0E-02
TIA	213	21,650	1.14	0.99	1.32	6.5E-02
Angina without revascularization	191	21,243	1.11	0.96	1.29	1.7E-01
Valvular disease	174	21,689	1.11	0.95	1.30	1.9E-01
Deep vein thrombosis	519	21,344	0.97	0.88	1.06	4.8E-01
Hemorrhagic stroke	214	21,649	0.97	0.84	1.11	6.3E-01
Pulmonary embolus	399	21,464	0.99	0.90	1.10	9.1E-01

Supplementary Table 5. Sensitivity analysis of adjudicated cancer outcomes among the subset of women genotyped with the Oncochip.

Outcome	Cases	Controls	Odds ratio	LCI	UCI	P value
Any cancer	4150	2808	0.96	0.91	1.01	0.1
Breast cancer	3734	3224	0.97	0.92	1.02	0.2
Lung cancer	167	6791	0.92	0.78	1.07	0.3

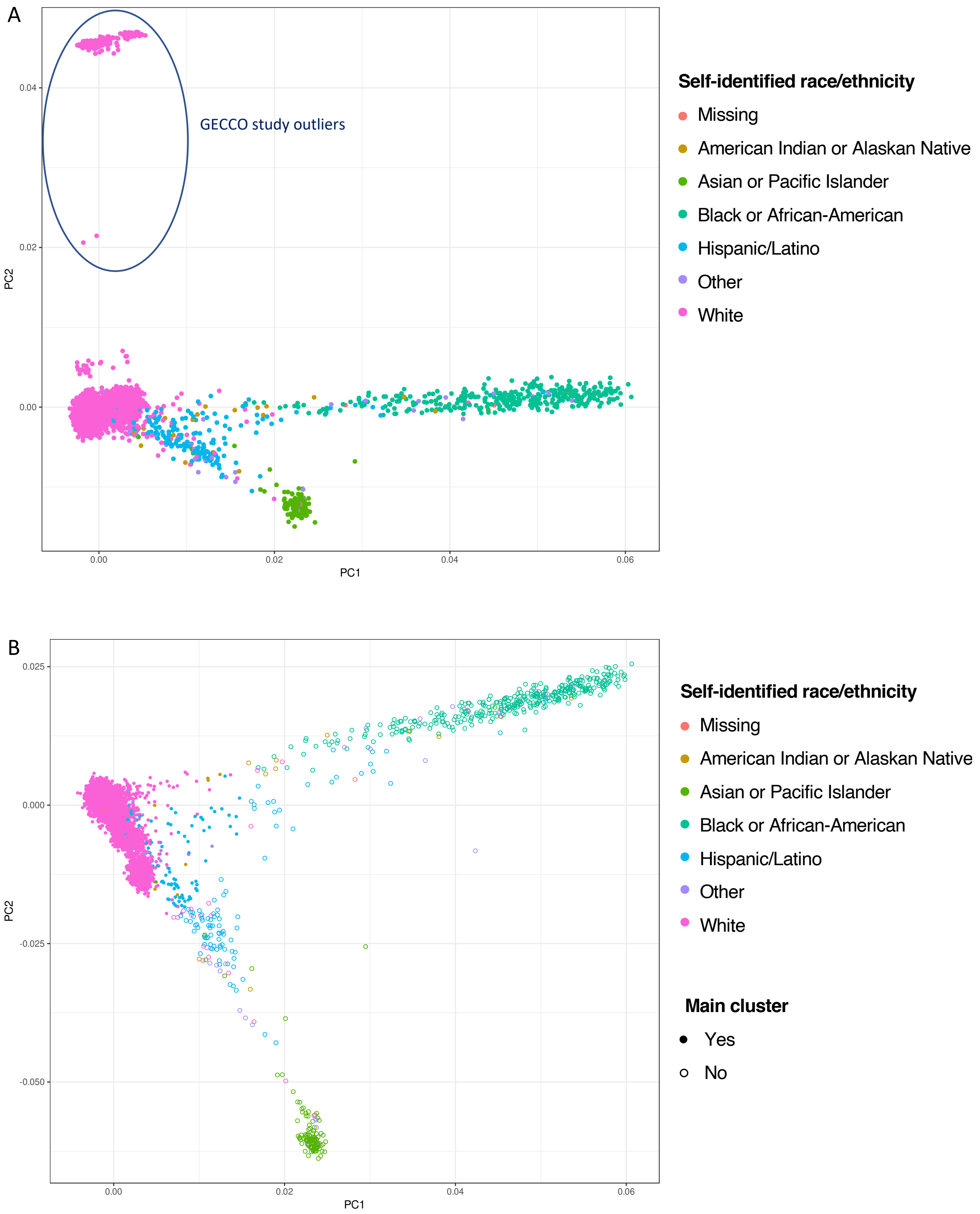

Supplementary Figure 1. Principal components analysis of the genotyped cohort. (A) In the initial analysis, 472 outliers from the GECCO study were identified. These subjects were removed. (B) Principal components were rerun. A cohort of 24,693 subjects (main cluster) were identified as having similar inferred genetic ancestry by the Mahalanobis distance method.

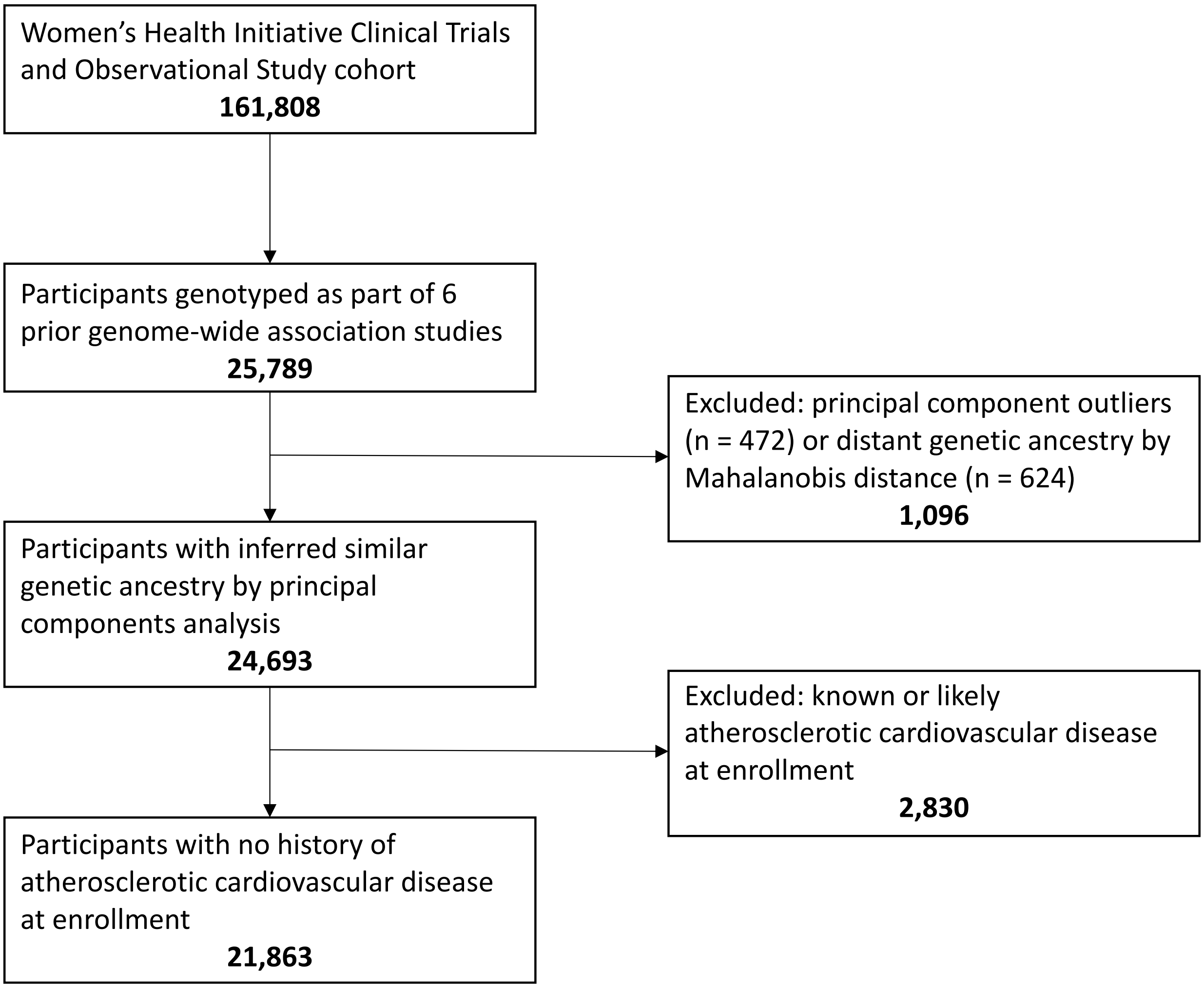

Supplementary Figure 2. Flow diagram of Women's Health Initiative subjects selected for this study.

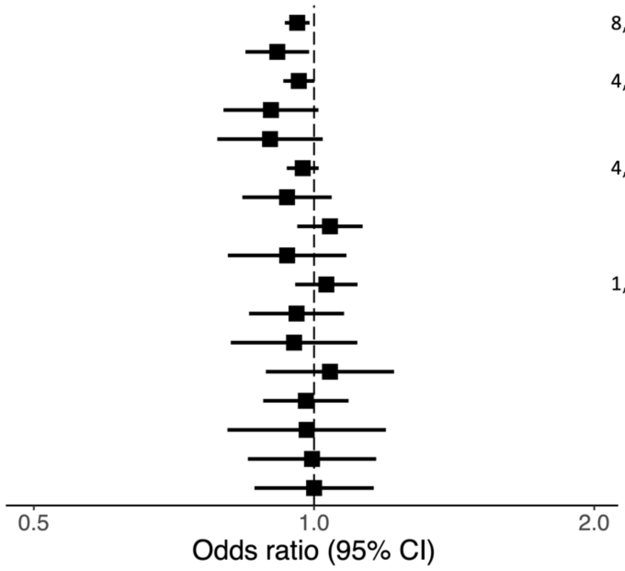

Outcome	Cases	Controls	OR (95% CI)	P value
Any cancer	8,294	13,569	0.96 (0.93-0.99)	7.7E-03
Lung cancer	696	21,167	0.91 (0.84-0.99)	2.4E-02
Breast cancer	4,928	16,935	0.96 (0.93-1.00)	5.4E-02
Breast cancer in situ	309	21,554	0.90 (0.80-1.01)	7.5E-02
Endometrial cancer	248	21,615	0.90 (0.79-1.02)	0.10
Invasive breast cancer	4,686	17,177	0.97 (0.93-1.01)	0.16
Pancreatic cancer	358	21,505	0.94 (0.84-1.04)	0.24
Colon cancer	910	20,953	1.04 (0.96-1.13)	0.34
Leukemia	195	21,668	0.94 (0.81-1.08)	0.37
Colon or rectal cancer	1,151	20,712	1.03 (0.95-1.11)	0.44
Lymphoma	307	21,556	0.96 (0.85-1.08)	0.47
Bladder cancer	172	21,691	0.95 (0.81-1.11)	0.54
Ovarian cancer	168	21,695	1.04 (0.89-1.22)	0.63
Melanoma	383	21,480	0.98 (0.88-1.09)	071
Kidney cancer	110	21,753	0.98 (0.81-1.19)	0.85
Rectal cancer	193	21,670	0.99 (0.85-1.17)	0.95
Other cancer	194	21,669	1.00 (0.86-1.16)	1.00

Supplementary Figure 3. Associations between polygenic risk for coronary artery disease and incident adjudicated cancers in the Women's Health Initiative. Outcomes with at least 100 incident cases were considered. Odds ratios are per 1 standard deviation increase in polygenic risk score. Error bars represent 95% confidence intervals.
